# Immature articular cartilage and subchondral bone covered by menisci are potentially susceptive to mechanical load

**DOI:** 10.1186/1471-2474-15-101

**Published:** 2014-03-26

**Authors:** Hirotaka Iijima, Tomoki Aoyama, Akira Ito, Junichi Tajino, Momoko Nagai, Xiangkai Zhang, Shoki Yamaguchi, Haruhiko Akiyama, Hiroshi Kuroki

**Affiliations:** 1 Department of Motor Function Analysis, Human Health Sciences, Graduate School of Medicine, Kyoto University, 53 Shogoin, Sakyo-ku, Kyoto 606-8507, Japan; 2 Department of Development and Rehabilitation of Motor Function, Human Health Sciences, Graduate School of Medicine, Kyoto University, Kyoto, Japan; 3 Department of Orthopaedic Surgery, Graduate School of Medicine, Kyoto University, Kyoto, Japan

**Keywords:** Articular cartilage, Uncovered region, Covered region, Superficial layer, Subchondral bone architecture

## Abstract

**Background:**

The differences of mechanical and histological properties between cartilage covered by menisci and uncovered by menisci may contribute to the osteoarthritis after meniscectomy and these differences are not fully understood. The purpose of this study is to investigate potential differences in the mechanical and histological properties, and in particular the collagen architecture, of the superficial cartilage layer and subchondral bone between regions covered and uncovered by menisci using immature knee.

**Methods:**

Osteochondral plugs were obtained from porcine tibial cartilage that was either covered or uncovered by menisci. Investigation of the thickness, mechanical properties, histology, and water content of the cartilage as well as micro-computed tomography analysis of the subchondral bone was performed to compare these regions. Collagen architecture was also assessed by using scanning electron microscopy.

**Results:**

Compared to the cartilage uncovered by menisci, that covered by menisci was thinner and showed a higher deformity to compression loading and higher water content. In the superficial layer of cartilage in the uncovered regions, collagen fibers showed high density, whereas they showed low density in covered regions. Furthermore, subchondral bone architecture varied between the 2 regions, and showed low bone density in covered regions.

**Conclusions:**

Cartilage covered by menisci differed from that uncovered in both its mechanical and histological properties, especially with regards to the density of the superficial collagen layer. These regional differences may be related to local mechanical environment in normal condition and indicate that cartilage covered by menisci is tightly guarded by menisci from extreme mechanical loading. Our results indicate that immature cartilage degeneration and subchondral microfracture may occur easily to extreme direct mechanical loading in covered region after meniscectomy.

## Background

In intact knee joints, tibial cartilage is divided into 2 regions: the cartilage covered by the menisci and that uncovered by menisci. Cartilage uncovered by menisci bears the body weight directly, whereas cartilage covered by menisci does so indirectly through the menisci. The pattern of loading during locomotion has a substantial influence on the general characteristic of the regional variation of cartilage [1]. Therefore, cartilage covered by menisci is thinner than cartilage uncovered by menisci [2]. However, it has been suggested that post-meniscectomy structural changes in the knee lead to alterations in the tibial contact area and cartilage thickness [3]. Kinematic changes have been implicated as a cause of osteoarthritis (OA) [4], and broaden areas of cartilage degeneration [5]. Recently, tibial cartilage covered by menisci may be damaged after meniscectomy exclusively [6]. Currently, these were thought to be because of increase contact stress following reduced contact area [3]. However, recent studies indicated that cartilage covered by menisci have different histological character compare to cartlilage uncovered by menisci [1,7,8]. Therefore, investigating the regional differences between cartilage covered by menisci and uncovered by menisci is important for understanding the mechanism of cartilage degradation after meniscectomy.

Meniscectomy have been performed not only to adult but also to children in such a case of discoid meniscus [9]. Caution is required when treating children because premature arthritis is common in patients who undergo total meniscectomy during childhood [9]. Previous study showed that injury to skeletally immature cartilage are vulnerable to load-induced injury than mature cartilage [10]. Therefore, understanding whether cartilage covered by menisci is susceptive to mechanical loading in immature knee is important for prevention of OA changes.

Evaluation of mechanical properties is important for investigating the cartilage degeneration process [11], and it is believed that changes in mechanical properties are superior to alterations in the composition of the cartilage matrix [12]. The mechanical properties of cartilage may vary according to whether it is covered or uncovered by menisci by the reason why mechanical environment differ in these regions [1,7,8]. Several studies showed that articular cartilage covered by menisci was stiffer [1,7] and thinner [1] compared to cartilage uncovered by menisci. These studies focused on only the mechanical properties and histological findings of the main components of the cartilage matrix, such as glycosaminoglycan and collagen, in regions covered and uncovered by menisci. However, the mechanical properties of cartilage are affected by not only proteoglycan and collagen content [13-15], but also water content [15] and collagen integrity [15,16]. Therefore, the reason why mechanical properties differ in regions covered by menisci and uncovered by menisci is still unknown.

Of each of the cartilage layers, the superficial layer of articular cartilage is thought to be particularly important in determining its mechanical properties [17]. Recent studies have reported that superficial collagen have a role for countervail to compression stress and shear stress [17,18]. Moreover, it is thought to have different collagen subtype, I, II, and III compare to middle layer, deep layer which have only type II collagen [19]. However, potential differences in collagen fiber structure between these two regions, especially in the superficial layer of cartilage, which affect cartilage mechanical properties, are yet to be determined.

Subchondral bone may have a substantial role in the OA process. In previous studies, knee radiographs have shown property changes and scleroses in the subchondral bone [20,21]. In recent studies, these subchondral bone changes have been evaluated using micro-computed tomography (micro-CT) in animal OA models [22-25] and human OA [26,27]. These studies have shown that the components of subchondral bone architecture, including bone mineral density (BV/TV), trabecular thickness (Tb.Th), and trabecular separation (Tb.Sp), were altered in OA and that these bone changes precede cartilage damage [28,29]. These subchondral bone architecture thought to be associated with local load magnitude during locomotion, a spatial shift in the contact area could place loads on a region of subchondral bone that may not adapt to the rapidly increased load after meniscectomy [30]. Understanding site-specific subchondral bone architecture is important because extreme mechanical load transferred to the osteoporotic subchondral bone may cause microfracture, which may lead to scleroses of the subchondral bone [20]. Although mechanical load transferred to the subchondral bone may differ between regions covered and uncovered by menisci, to our knowledge, no study has focused on the differences in subchondral bone architecture between these regions.

Thus, the main aim of the present study was to investigate the mechanical and histological properties, particularly the collagen fiber structure of the superficial cartilage layer and aspects of subchondral bone architecture that may differ between cartilage covered and uncovered by menisci using immature knee. Our hypothesis is 1) the mechanical properties of the cartilage differ between in regions covered and uncovered by menisci, 2) collagen fiber of the superficial zone shows low density in cartilage covered by menisci, 3) subchondral trabecular bone architecture shows low density in cartilage covered by menisci.

## Method

### Sample preparation

**Figure 1:**
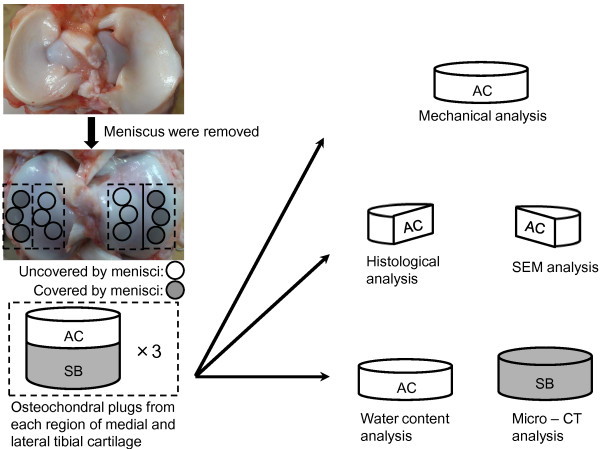
**Preparation of samples in regions covered and uncovered by menisci.** A total of 12 osteochondral plugs were obtained from the medial and lateral tibial cartilages. Three plugs obtained from each region were used for mechanical analysis, histological analysis, scanning electron microscopic (SEM) analysis, determination of water content ratio, and micro-computed tomography (CT) analysis. The cartilage layer alone was used for mechanical analysis, histological analysis, SEM analysis, and water content analysis. The subchondral bone alone was used for micro-CT analysis. *AC* articular cartilage, *SB* subchondral bone.

Articular cartilage was obtained from seven 6-month-old porcine knees within 12 h of sacrifice as retailing items for consumption. No approval was required from the Animal Committee of Kyoto University, because the animals were sacrificed at a slaughterhouse for commerce and the porcine knees were purchased from Aota-Chikusan Company (Kyoto, Japan). After the joints were opened, 12 osteochondral plugs were obtained from the medial and lateral tibial cartilage, which were covered or uncovered by menisci [1,7,8] (Figure 1). Only sites with a macroscopically normal articular cartilage were included in this study [12]. Three plugs obtained from each region were used for mechanical analysis, histological analysis, scanning electron microscopy (SEM) analysis, determination of water content ratio, and micro-CT analysis. For mechanical analysis, subchondral bone was removed from osteochondral plug and only cartilage layer was used. The subchondral bone was removed from 1 osteochondral plug and the remaining cartilage was divided into 2 pieces. One was used for histological analysis and the other for SEM analysis. The remaining part of the osteochondral plug was divided into cartilage and subchondral bone plugs; the cartilage was used for determination of water content ratio and the subchondral bone was used for micro-CT analysis.

### Mechanical analysis of cartilage

Before mechanical analysis, cartilage thickness was measured using a stereoscopic microscope (Multi viewer system, Keyence, Osaka, Japan) [12,17]. Cartilage thickness was defined as the distance between the cartilage surface and osteochondral junction. For each sample, a mean thickness was calculated from four measurements taken around the circumference of the plug [12,17]. After this, indentation test [1] was applied using a mechanical testing instrument with the use of a 0.5 mm diameter indenter (Autograph AG-X, Shimazu, Kyoto, Japan). Each sample was compressed uniaxially in a testing chamber filled with phosphate buffered saline (PBS) at room temperature. A pre-load of 0.01 N was applied and allowed to equilibrate for 100 seconds. Then, loading was applied at a strain rate of 0.005 mm/s up to 0.01 N and maintained for 10 min. Preliminary reports revealed that a time of 10 min ensured sufficient relaxation to allow the samples to fully equilibrate [31]. Two features were determined from this indentation test: creep (mm), creep normalized to cartilage thickness (creep/thickness) [1].

### Histological analysis of cartilage

For histological analysis, specimens were fixed with 4% paraformaldehyde. The fixed samples were decalcified with 10% ethylenediaminetetraacetic acid at 4°C for 10 days. After decalcification, the specimens were embedded in paraffin. Specimens were then sliced in 6-μm serial sections and stained with safranin O and fast green staining for assessment of proteoglycan distribution as well as with picrosirius red staining for measurement of collagen orientation under a polarizing microscope (PLM) (Eclipse 80i, Nikon, Tokyo, Japan).

Immunohistochemical localization of type II collagen was also performed. Deparaffinized sections were treated with 0.3% hydrogen peroxide to reduce endogenous peroxidase activity. Then, the sections were treated with 1.25% hyaluronidase (Sigma-Aldrich Co., St Louis, MO, USA) in PBS for 60 min at room temperature. After rinsing thrice with PBS for 5 min, non-specific binding sites were blocked by treating the sections with 2% normal horse serum for 60 min at room temperature. Subsequently, the sections were treated with anti-type II collagen (diluted 1:100; Fine Chemical Co., Toyama, Japan) and further incubated overnight at 4°C. Sections were then rinsed in PBS and treated with horse biotinylated anti-mouse IgG (diluted 1:200; Vector Laboratories, Burlingame, CA, USA) for 30 min at room temperature. Detection was performed using the streptavidin–biotin–peroxidase complex technique with an Elite ABC kit (diluted 1:100; Vector Laboratories). Immunoreactivity was visualized by incubation with diaminobenzidine solution (Vector Laboratories) for 5 min followed by counterstaining with hematoxylin. The primary antibody was not added to negative controls.

In order to analyse these histologically stained sections quantitatively, under microscopy and standardized condition, pictures of TIFF images were taken at magnification ×40 and TIFF images were converted to greyscale (white, 255; black, 0) with the images software. These converted images were analysed for the matrix staining intensity levels with depth from the cartilage surface. Rectangles (30 μm deep and 400 μm long) were superimposed in each depth from the cartilage surface to osteochondral region (0, 25, 50, 75, 100% of depth [17,32]. The matrix staining intensity was evaluated at 10 evenly spread point in the each rectangle respectively.

### SEM analysis of cartilage

SEM analysis was performed to assess the ultramicrostructure of collagen fibers in articular cartilage by using a SEM system (H-7650, Hitachi, Tokyo, Japan). Samples were fixed with 4% paraformaldehyde–2% glutaraldehyde solution. After washing in PBS, the specimens were fixed with osmic acid for 90 min at room temperature. Samples were then dehydrated with ethanol. Tert-butyl alcohol was used for freeze-drying. Specimens were mounted on aluminum stubs and coated with platinum/palladium. The collagen fiber in superficial layer, middle layer, deep layer of cartilage in regions covered and uncovered by menisci was observed under SEM.

### Water content of cartilage

Water content was determined by weighing the cartilage regions covered and uncovered by menisci before and after drying, in 60°C for 12 h. Water content was expressed as a percentage of the wet weight [33].

### Micro-CT analysis of subchondral bone

A high-resolution micro-CT scanner (SMX-100CT, Shimazu) was used to determine the BV/TV, Tb.Th, and Tb.Sp of the subchondral bone from the medial and lateral tibial plateaus under the regions covered and uncovered by menisci. A columnar region measuring 6 mm in diameter and 500 μm in height and located 1 mm beneath the osteochondral junction was scanned, in order to exclude small bone fragments that were created while preparing the bone samples. BV/TV, Tb.Th, and Tb.Sp were calculated based on the 3D image data sets obtained using a computer software (Amira5.4, Visage, Berlin, Germany).

### Statistical analysis

The software program SPSS for Windows (SPSS Japan Inc., Tokyo, Japan) was used for statistical analysis. The statistical significance of any differences between groups was determined using *t*-test for unpaired data sets and a one-way analysis of variance (ANOVA), followed by Tukey’s test for multiple comparisons. Descriptive statistics were calculated as means and 95% confidence intervals (CI). In all cases, *P* < 0.05 was considered significant. The statistical power to detect a significant difference between the groups was calculated.

## Results

### Mechanical properties of cartilage

**Figure 2:**
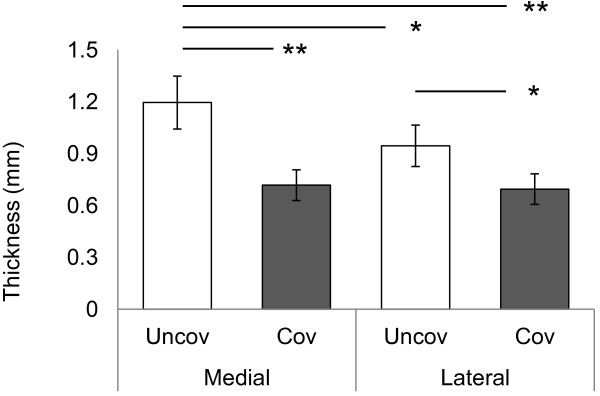
**Thickness of the articular cartilage in the regions covered and uncovered by menisci.** Bars show the mean ± 95% confidence interval. *P < 0.05; **P < 0.01.

**Figure 3:**
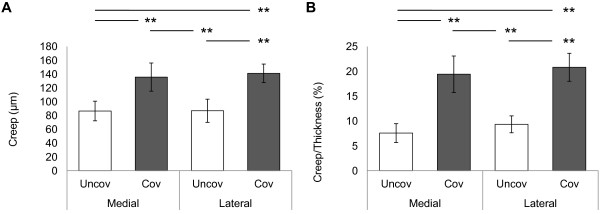
**Mechanical analysis of cartilage in regions covered and uncovered by menisci.** Creep of the medial and lateral tibial cartilage **(A)**. Creep normalized to cartilage thickness (Creep/Thickness) of the medial and lateral tibial cartilage **(B)**. Bars show the mean ± 95% confidence interval. **P < 0.01.

No osteochondral plugs showed degenerative change at sample preparation. The average cartilage thickness was significantly thinner in medial and lateral covered regions (Figure 2). Creep were significantly larger in the covered regions in medial and lateral tibial cartilage (Figure 3A). Creep normalized to cartilage thickness were also significantly larger in the covered regions in medial and lateral tibial cartilage (Figure 3B). Retrospectively, the power of ANOVA test was 1.000 for average cartilage thickness, 0.996 for creep and 1.000 for creep normalized cartilage thickness, respectively.

### Histological findings in cartilage

**Figure 4:**
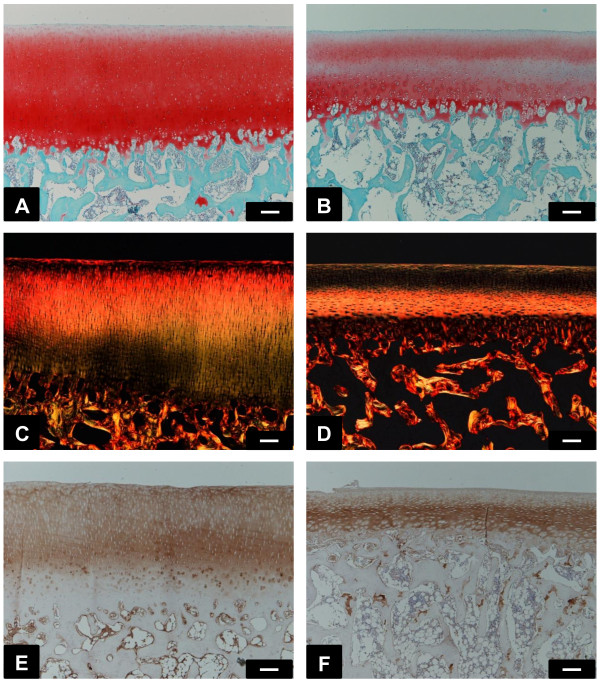
**Photographs of the tibial cartilage uncovered by menisci (A, C, E) and of those covered by menisci (B, D, F).** Safranin O and fast green staining **(A, B)**. Picrosirius red staining **(C, D)**. Type II collagen **(E, F)**. *Scale bars* 100 μm **(A–F)**.

Safranin O staining revealed a high proteoglycan content in medial and lateral regions uncovered by menisci (Figure 4A, B). The pattern of picrosirius staining was differ between region covered and uncovered by menisci (Figure 4C, D). The presence of non-aligned fibers from the surface to the middle layer was observed in cartilage covered by menisci.

In immunohistochemical observations of type II collagen in the cartilage, staining intensity and the expression pattern of type II collagen differed between the two regions. In the region uncovered by menisci, type II collagen was expressed in all layers (Figure 4E), whereas expression was less pronounced in the superficial zone in the covered regions (Figure 4F).

**Figure 5:**
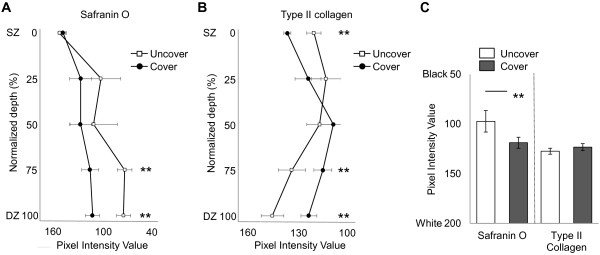
**Matrix staining of with depth from the articular surface of (A) safranin O stain and (B) type II collagen srain.** The average of these intensity value **(C)**. Bars show the mean ± 95% confidence interval. **P < 0.01.

Significantly higher light intensity was measured in the safranin O stained sections of region covered by menisci especially in deep layer (Figure 5A), indicating less intense staining in this region of the cartilage. Although there were no significant differences in average of intensity of type II collagen staining (Figure 5C), significantly higher light intensity was measured in superficial layer of region covered by menisci (Figure 5B). Retrospectively, the power of *t*-test was 0.890 for safranin O staining and 0.367 for type II collagen, respectively.

### SEM observations

**Figure 6:**
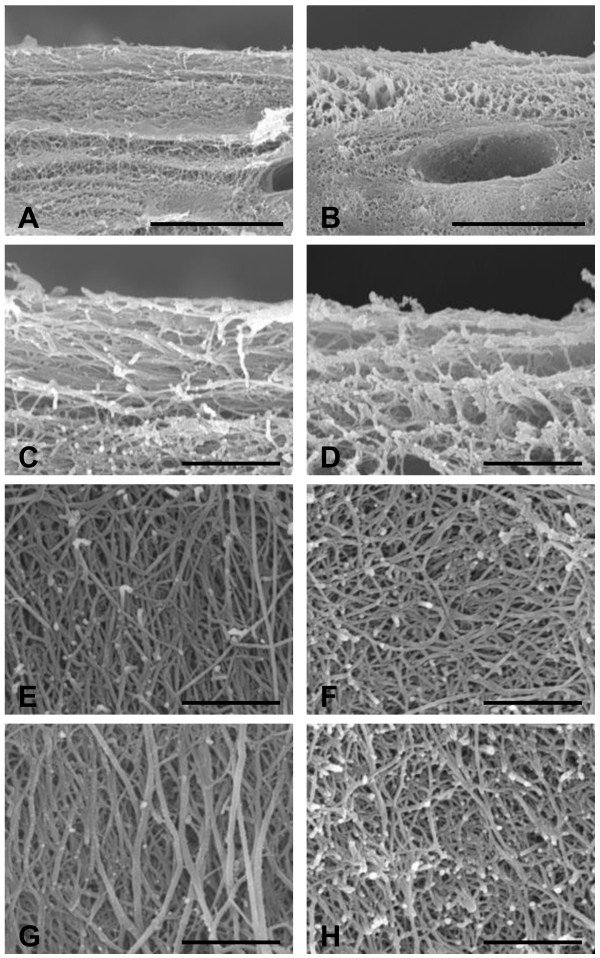
**Scanning electron microscopic (SEM) photographs of the tibial cartilage.** Collagen fibers in regions uncovered by menisci **(A, C, E, G)** and in those covered by menisci **(B, D, F, H)**. Superficial layer **(A–D)**. Middle layer **(E–F)**. Deep layer **(G–H)**. *Scale bars* 10 μm **(A, B)** and 2 μm **(C–H)**.

In the superficial layer of cartilage in the regions uncovered by menisci, collagen fiber orientation was parallel and showed a high degree of density (Figure 6A, C). On the other hand, in the covered regions, collagen fiber orientation was parallel but slightly random and had a low degree of density (Figure 6B, D). In the middle (Figure 6E) and deep layers (Figure 6G) of the cartilage, collagen fiber orientation in the regions uncovered by menisci was perpendicular to the cartilage surface. On the other hand, in the covered regions, collagen fiber orientation was not perpendicular but random in the middle (Figure 6F) and deep layers (Figure 6H).

### Water content of cartilage

**Figure 7:**
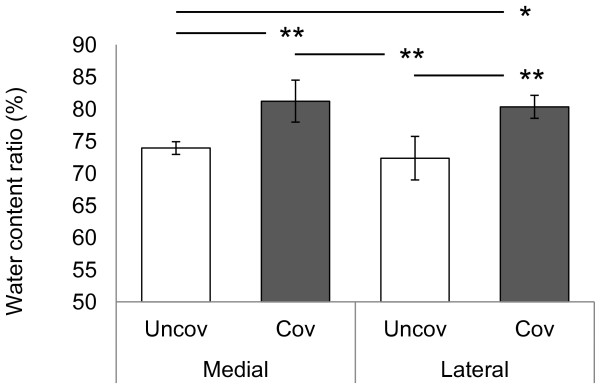
**Water content ratio of articular cartilage in regions covered and uncovered by menisci.** Bars show the mean ± 95% confidence interval. *P < 0.05; **P < 0.01.

Results of water content ratio of cartilage were shown Figure 7. Articular cartilage covered by menisci showed a higher water content ratio in medial tibial cartilage (medial uncovered region: mean, 73.9%; 95% CI, 72.9–74.9 and medial covered region: mean, 81.2%; 95% CI, 78.0–84.5) and lateral tibial cartilage (lateral uncovered region: mean, 72.3%; 95% CI, 69.0–75.7 and lateral covered region: mean, 80.3%; 95% CI, 78.5–82.1). Retrospectively, the power of ANOVA test was 1.000 for water content of cartilage.

### Micro-CT analysis of subchondral bone

**Figure 8:**
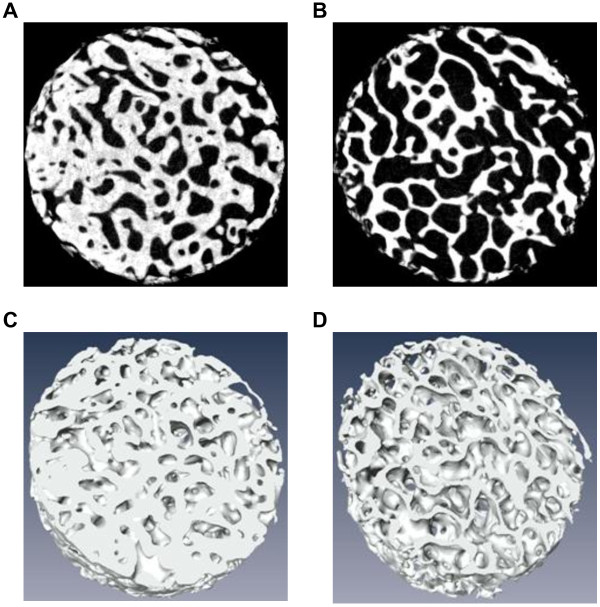
**Subchondral bone architecture in regions uncovered by menisci (A, C) and in those covered by menisci (B, D).** Two-dimensional images of subchondral bone **(A, B)**. Three-dimensional images of subchondral bone **(C, D)**.

**Figure 9:**
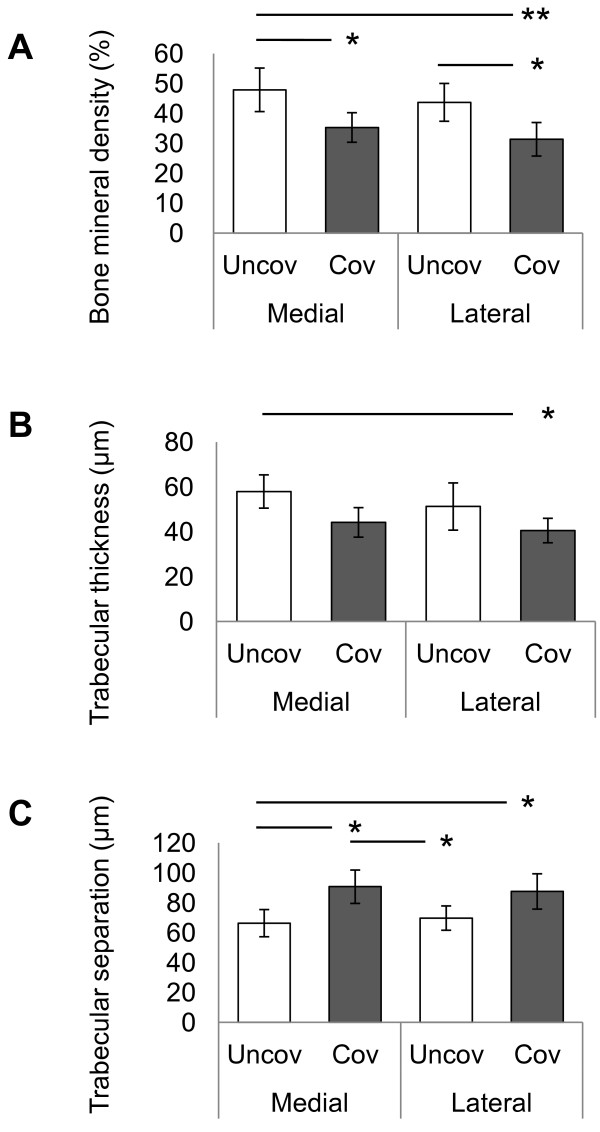
**Micro-computed tomographic analysis in regions covered and uncovered by menisci.** Bone mineral density (BV/TV) of the medial and lateral tibial subchondral bone **(A)**. Trabecular thickness (Tb.Th) of the medial and lateral tibial subchondral bone **(B)**. Trabecular separation (Tb.Sp) of the medial and lateral tibial subchondral bone **(C)**. Bars show the mean ± 95% confidence interval. *P < 0.05; **P < 0.01.

Images of the subchondral bone architecture in each region are shown in Figure 8A-D. BV/TV was significantly lower in the covered regions of medial (Figure 9A) (medial uncovered region: mean, 47.9%; 95% CI, 40.6–55.2 and medial covered region: mean, 35.0%; 95% CI, 30.4–40.3) and lateral (lateral uncovered region: mean, 43.7%; 95% CI, 37.4–50.1 and lateral covered region: mean, 31.4%; 95% CI, 25.8–37.0). Tb.Th was significantly thinner only in the covered regions of lateral tibial cartilage than the uncovered regions of medial (Figure 9B) (medial uncovered region: mean, 57.9 μm; 95% CI, 65.4–50.5 and lateral covered region: mean, 40.5 μm; 95% CI, 46.0–35.1). Tb.Sp was significantly larger in the covered regions of medial than uncovered region of medial and lateral (Figure 9C) (medial uncovered region: mean, 66.3 μm; 95% CI, 57.2–75.4, medial covered region: mean, 90.7 μm; 95% CI, 79.6–101.8, lateral uncovered region: mean, 69.7 μm; 96% CI, 61.5–77.8). Tb.Sp in the covered region of lateral (lateral covered region: mean, 87.5 μm; 95% CI, 75.7–99.3) was larger than uncovered region of medial. Retrospectively, the power of ANOVA test was 0.918 for BV/TV, 0.751 for Tb.Th, and 0.901 for Tb.Sp, respectively.

## Discussion

In this study, we compared the mechanical and histological properties, water content, and SEM findings of cartilage and micro-CT findings of subchondral bone architecture between the regions covered and uncovered by menisci. Our results revealed differences in the mechanical properties and structures of articular cartilage and subchondral bone between the 2 regions.

We found that the cartilage was thinner in the regions covered by menisci (Figure 2). This result is consistent with that of previous studies [16,33]. A report, which showed that the thickness of the cartilage covered by menisci changed after meniscectomy [33], indicated that menisci protect the cartilage covered by menisci from extreme direct mechanical loading.

The present study showed that the mechanical properties of the tibial articular cartilage differed between the regions covered and uncovered by menisci (Figure 3). Previous studies [8] have reported that the tibial plateau cartilage covered by menisci showed higher deformity to compression load than the cartilage uncovered by menisci and the result of the present study is consistent with previous finding. Although it is unclear what these differences in mechanical properties between the 2 regions may mean, our findings, showing low density of collagen in the superficial layer (Figure 6) may contribute to mechanical properties. Superficial layer of cartilage is component 10-20% of cartilage thickness [34] and it have ability to maintain fluid load support to compression loading [15]. In our study, creep normalized to cartilage thickness from indentation method is almost 20% in cartilage covered by menisci, nearly twice compare to cartilage uncovered by menisci. Therefore, our indentation methods may mainly reflects superficial structure and partly middle and deep layer structure. Water content is related to permeability [35,36], which may indicate matrix resistance to pressure-induced fluid flow [37], fixed charge density [38], and superficial collagen integrity [18,39]. Previous studies have determined high water content in degenerated cartilage and have reported that this cartilage shows greater deformation of the solid matrix [36,37]. Our findings showing low proteoglycan content (Figures 4 and 5), low collagen density in the superficial layer (Figure 6), and high water content in covered regions (Figure 7) suggest that covered regions have lower matrix resistance to mechanical load, and that the cartilage in covered regions show high deformity to compression load.

In our study, attention should be paid for interpretation of the results. Because, previous studies showed growth effects on the collagen structure [40] and collagen content can double during the maturation process [41]. In addition, the organization of the collagen fibers progressed from a somewhat random collagen meshwork in immature cartilage to a more organized arrangement with maturation [42]. Previous studies showed that immature cartilage have a vulnerability of soft superficial layer to compressive injury [43]. Therefore, the histology and ultrastructure of collagen fibers by SEM observations from this study might not be translated to mature sample, additionally to human due to of anatomical differences [44]. However, our findings, which immature cartilage covered by menisci have low cartilage matrix, might partly support early degenerative changes in cartilage after total meniscectomy in child knee [9].

In OA, subchondral scleroses are one of the earliest radiographic changes detected. This change often occurs before joint space narrowing of knee OA [20,21]. Once scleroses occur, bone volume and bone density increase, thus causing thickening of the subchondral bone [20,45]. This change may be due to bone remodeling in response to repetitive microfracture by overloading [46,47]. We observed that bone density was low in the covered regions (Figure 9A, B); this implies that subchondral bone in covered regions is weak with regards to excessive mechanical load, and that excessive repetitive mechanical load is the cause of microfractures and scleroses in the covered regions. Our results showing thinner cartilage, low collagen density, and low proteoglycan content in the superficial layer of cartilage as well as high water content in covered regions suggest that the cartilage in covered regions is susceptible to excessive mechanical load, and that extreme mechanical load is thereby transmitted to the subchondral bone more easily. Although this study was not performed in humans, the weakness seen in the subchondral bone together with the weakness of the superficial layer of cartilage in covered regions suggests that covered regions are more susceptible to subchondral microfracture and subchondral bone scleroses. Although the weakness to excessive mechanical load seen in the superficial layer of cartilage and subchondral bone in the covered regions is compensated for by the menisci, i.e., the meniscus–cartilage–subchondral bone unit receives mechanical load, this compensation does not occur after meniscectomy. Therefore, it is not the meniscus–cartilage–subchondral bone unit but the cartilage–subchondral bone unit in the covered regions that receives the mechanical load in this situation. After meniscectomy, the contact area between the femur and tibia changes [3] and contact mechanical load between the femur and tibia increases [3]. Therefore, meniscectomy leads to subchondral bone microfracture [48,49], subchondral bone scleroses in covered regions [30], and cartilage degeneration [30,33,50]. Our findings strongly support the reports that suggest that subchondral bone scleroses and cartilage degeneration often occur after meniscectomy.

This study has several potential limitations. First, we used immature, 6-month-old porcine samples. There may have been some differences in collagen architecture and proteoglycan content as compared to mature porcine [51] or other species [43,52], therefore our finding might not be translated directly to mature samples or to humans. Second, we obtained from tibial cartilage covered by menisci and uncovered by menisci, however the menisci are known to shift slightly during joint movement. Some of the region could receive mechanical loading both meniscus-uncovered and meniscus-covered, therefore the results cannot still be trusted to depend purely on the menisci, but instead they may reflect mechanical adaptation [53]. Third, we evaluated collagen density in superficial layer of cartilage using SEM system. Although SEM observation indicate lower collagen density in cartilage covered by menisci, which partly support immunohistochemical finding of type II collagen, this is a limitation preventing of results with statistical support because SEM observation is a qualitative evaluation. Fourth limitation is the technique used to measure mean cartilage thickness. In this study, we evaluated mean cartilage thickness of samples obtained from the osteochondral plugs by using a stereoscopic microscope. Thus, the mean cartilage thicknesses of the regions covered and uncovered by menisci may have differed a little from the actual cartilage thickness. Fifth, we evaluated bone architecture by determining BV/TV, Tb.Th, and Tb.Sp on the basis of the findings of micro-CT analysis; however, these measurements do not always provide proper reflections of the mechanical properties of subchondral bone [54].

## Conclusions

In conclusion, our study confirmed that the cartilage covered by menisci differs from that uncovered with respect to its cartilage thickness, mechanical properties, proteoglycan content, water content, and collagen architecture. For the first time, we found that the collagen architecture of the superficial zone in covered regions showed a low density upon SEM analysis, and that the architecture of the subchondral bone covered by menisci showed a low bone density and thinner trabecular bone upon micro-CT analysis. These findings indicate that immature cartilage in covered regions is potentially susceptive to excessive mechanical load. This weakness may be compensated for by the menisci, which overcome mechanical loads in normal conditions. Therefore, our findings support the view that immature cartilage degeneration and subchondral microfracture occur easily after meniscectomy, which exposes the previously covered regions of the cartilage to direct mechanical load. Further study is needed to assess how structural changes and degeneration occur more easily in cartilage covered by menisci after meniscectomy in vivo.

## Abbreviations

OA: Osteoarthritis; micro-CT: Micro-computed tomography; BV/TV: Bone mineral density; Tb.Th: Trabecular thickness; Tb.Sp: Trabecular separation; SEM: Scanning electron microscopy; PBS: Phosphate buffered saline; PLM: Polarizing microscope.

## Competing interests

The authors declare that they have no competing interests.

## Authors’ contributions

HI and HK designed the study, had full access to all of the data in the study, analyzed the data, take responsibility for the integrity of the data and the accuracy of the data analysis, interpreted the data, and drafted the manuscript. TA and HA helped to design the study and contributed to the overall project management. HI, AI, MN, SY and JT contributed for sample preparation. HI and XZ performed mechanical testing. HI, SY and JT participated in the analysis of the histology and AI helped to observation ultrastructure of collagen fiber by SEM system. HI, MN performed micro-CT analysis of subchondral bone. HI interpreted the data, and HI and HK drafted the manuscript. HI was responsible for overall project management and designed the study. All authors critically revised the manuscript and read and approved the final manuscript for publication.

## Pre-publication history

The pre-publication history for this paper can be accessed here:

http://www.biomedcentral.com/1471-2474/15/101/prepub
